# Lunasin Inhibits Cell Proliferation via Apoptosis and Reduces the Production of Proinflammatory Cytokines in Cultured Rheumatoid Arthritis Synovial Fibroblasts

**DOI:** 10.1155/2015/346839

**Published:** 2015-01-27

**Authors:** Shaohui Jia, Shufang Zhang, Hong Yuan, Ning Chen

**Affiliations:** ^1^College of Health Science, Hubei Provincial Collaborative Innovation Center for Exercise and Health Promotion, Wuhan Sports University, Wuhan 430079, China; ^2^Graduate School, Wuhan Sports University, Wuhan 430079, China; ^3^College of Sports Science and Technology, Wuhan Sports University, Wuhan 430205, China

## Abstract

Lunasin, a peptide with 43 amino acid residues and initially isolated and identified in soybean cotyledon, has gained extensive attention due to its anti-inflammatory and anticancer properties. However, its treatment efficacy on rheumatoid arthritis (RA) and corresponding mechanisms have not been reported. Herein, the synovial fibroblasts harvested and isolated from patients with RA were treated with lunasin at various concentrations to examine the proliferation, apoptosis status, and corresponding cell cycle of cultured RA synovial fibroblasts. Meanwhile, the underlying mechanisms of lunasin for RA treatment are explored through Western blot, real-time PCR, ELISA, and luciferase reporter assays. Lunasin significantly inhibited the proliferation and induced the apoptosis of cultured RA synovial fibroblasts. In addition, lunasin reduced the production of interleukin-6 (IL-6), IL-8, and matrix metalloproteinase-3 (MMP-3) and suppressed the activation of NF-*κ*B in cultured RA synovial fibroblasts but did not reveal obvious modulation on the secretion and gene expression of MMP-1. Therefore, lunasin will have promising potential as a novel nutritional supplement or drug candidate for RA due to its potency of suppressing synovial cell proliferation and decreasing the production of proinflammatory cytokines and MMPs in synovial cells.

## 1. Introduction

Rheumatoid arthritis (RA) is a systematic inflammatory disease and afflicts 0.5–1.0% of population all over the world [1, 2]. RA is characterized by inflammatory changes of synovial tissues and the formation of rheumatoid pannus that results in the erosion of adjacent cartilage and subchondral bone, thus subsequently causing irreparable joint destruction. Pathological studies show that synovial fibroblasts play a critical role in the pathogenesis of RA [3]. The markedly increases of synovial fibroblasts in RA have been reported in previous studies [4] and the proliferation of synovial fibroblasts has been thought to contribute to the formation of rheumatoid pannus [5]. Moreover, RA synovial fibroblasts can spontaneously secrete numerous proinflammatory cytokines such as interleukin-6 (IL-6) and IL-8 and matrix metalloproteinases (MMPs) including MMP-1 and MMP-3, which plays an important role in progressive destruction of articular cartilage and bone [6–9]. Currently, many natural compounds such as celastrol [10], sinomenine [11], and curcumin [12] derived from plants or plant extracts such as* Periploca sepium* extract [13] and* Tripterygium wilfordii* Hook. F. extract [14] have reported to treatment RA through suppressing the expression of proinflammatory cytokines or mediators, adhesion molecules and MMPs in synovial fibroblasts, the peptide-based agents from natural plants for inhibiting these cell signaling molecules or cell signaling pathways in preclinical or clinical investigations are less reported.

Lunasin is a 43-amino acid peptide isolated and identified from soybean and other plant sources [15]. Lunasin contains nine aspartic acid residues on its carboxyl terminal that have been found to be responsible for its antimitotic effect [16]. It also contains a cell adhesion motif composed of arginine-glycine-aspartic acid (RGD) residues to allow the attachment to the extracellular matrix and a predicated helix with structural homology to a conserved region of chromatin-binding proteins [17]. Earlier* in vitro* studies have shown that lunasin can exert chemopreventive properties in mammalian cells elicited by chemical carcinogens and viral oncogenes [18, 19]. Moreover, lunasin has been reported to reduce the tumor incidence of skin cancer and breast cancer in mouse models [20, 21]. Recent studies have begun to unmask the antioxidant and anti-inflammatory potentials of lunasin through inhibiting different inflammatory mediators in macrophage cell line RAW 264.7 [22, 23]. The pathogenesis of RA is mostly similar as tumors and mainly characterized by inflammatory change and the proliferation of synovial fibroblasts. Therefore, we speculate that lunasin may be benefit for RA because of its anti-inflammatory potency. In order to explore the treatment efficacy of lunasin for RA and underlying mechanisms, we investigated the effect of lunasin on the progression of RA using human synovial fibroblasts from knee joint of patients with RA as an* in vitro* model, which will provide the promising potential or treatment strategy of lunasin for RA as a novel supplement and drug candidate.

## 2. Materials and Methods

### 2.1. Polypeptide and Reagents

The polypeptide lunasin was synthesized by Senggong Company (Shanghai, China). 3-(4,5-Dimethylthiazol-2-yl)-2,5-diphenyltetrazolium bromide (MTT), propidium iodide (PI), and Triton X-100 were purchased from Sigma Chemical Company (St. Louis, MO, USA). The Annexin V-fluorescein isothiocyanate (FITC)/PI apoptosis detection kit was obtained from Beyotime Institute of Biotechnology (Shanghai, China). All primary antibodies were purchased from Santa Cruz Biotechnology (Santa Cruz, CA, USA).

### 2.2. Isolation and Culture of Synovial Cells

Synovial tissues were harvested from six patients undergoing knee replacement surgeries owing to their RA in Tongji Hospital (Wuhan, China). The patients were provided with the informed consent for research and the experimental protocols were reviewed and proved by Institutional Review Board at Tongji Hospital, Huazhong University of Science and Technology. The scores of all patients are higher than 7.

Synovial fibroblasts were isolated by sequential digestion of the dissected synovial tissues with type I collagenase and cultured in Dulbecco Modified Eagle Medium (DMEM) (Gibco, Grand Island, NY, USA) at 37°C in a humidified atmosphere with 5% CO_2_, supplemented with 10% (v/v) fetal bovine serum (FBS), 100 U/mL penicillin, and 100 mg/L streptomycin.

### 2.3. Cytokine Assay

Cells (1 × 10^6^ cells/well) were plated in 6-well cell culture plates overnight and then incubated with lunasin at designed concentrations (0, 10, 50, 100, and 200 *μ*M) for 72 h. After treatment, cell supernatants were collected to analyze the secretion of cytokines (IL-6, IL-8, MMP-1, and MMP-3) using enzyme linked immunosorbent assay (ELISA) kit (R&D Systems Inc., Minneapolis, MN, USA).

### 2.4. Cell Proliferation Assay

For cell proliferation assay, synovial fibroblasts were plated in 96-well plates at a density of 2.5 × 10^3^ cells per well and allowed to synchronize by incubation in serum-free DMEM medium for 48 h. Then, the cell culture medium was replaced by normal DMEM medium containing lunasin at varying concentrations (0, 10, 50, 100, and 200 *μ*M). Cells were counted at 0, 24, 48, and 72 h, and cell number was evaluated by crystal violet staining as described as follows: cells were fixed with 1% glutaraldehyde and stained with 0.1% crystal violet. The unbound dye was dissolved with 0.2% Triton X-100. Light enhancement linearly correlated with the cell number was analyzed at 570 nm using a fluorescence microplate reader (Tecan Sunrise, Salzburg, Austria).

### 2.5. Cell Cycle Analysis

The distribution of cell cycle was detected by flow cytometry according to the manufacturer's protocol. Briefly, synovial fibroblasts were seeded in 6-well plates at a density of 1 × 10^6^ cells/well. After adhesion, cells were starved for 48 h in serum-free DMEM medium. Then, the medium was replaced by fresh normal DMEM medium in the presence or absence of lunasin. After incubation for another 48 h, the cells were collected and fixed in 70% ice-cold ethanol. The cell samples were resuspended in 200 *μ*L of solution containing 50 *μ*g/mL PI, 0.1 mg/mL RNase A, and 0.1% Triton X-100 and then incubated at 37°C for 30 min. Finally, the cell cycle analysis was performed on a flow cytometer (Beckman Coulter, Brea, CA, USA).

### 2.6. Apoptosis Analysis

Synovial fibroblasts in 6-well plates (1 × 10^6^ cells/well) were treated with lunasin at various concentrations (0, 10, 50, 100, and 200 *μ*M) for 48 h, and 60 *μ*M H_2_O_2_ was used to induce apoptosis as the positive control. At the end of treatment, the cells were collected and washed with PBS for three times. Then, the cells were resuspended with 195 *μ*L of FITC-binding buffer and incubated with 5 *μ*L of Annexin V-FITC for 15 min at room temperature in a dark environment. After washed with PBS, the cells costained with PI solution were subjected to flow cytometric analysis.

### 2.7. RT-PCR Analysis

Total RNA was isolated using TRIzol reagent (Invitrogen, Carlsbad, CA, USA) and reversely transcribed to cDNA using a RevertAid cDNA Synthesis Kit (Fermentas International Inc., Vilnius, Lithuania) according to the manufacturer's instructions. The primer sequences and reaction conditions of IL-6, IL-8, MMP-1, MMP-3, and GAPDH were shown in Table 1. The PCR products were evaluated by 2% agarose gel electrophoresis.

### 2.8. Western Blot Analysis

After treatment, cell samples were lysed in 20 *μ*L of cell lysis buffer containing 1 mm phenylmethanesulfonyl fluoride (PMSF). The extracted proteins were separated by 17% SDS-PAGE and transferred to PVDF membranes by 2 h electroblotting. Blots were blocked in 5% nonfat dry milk for 1 h at room temperature and then incubated at 4°C overnight with primary antibodies. Membranes were washed with TBS containing 0.05% Tween-20 (TBS-T buffer) for 3 times and then incubated with horseradish peroxidase-conjugated secondary antibodies at 37°C for 1 h. Finally, the blots were developed with the enhanced chemiluminescence (ECL) kit (Pierce Biotechnology, Rock-ford, IL, USA).

### 2.9. Luciferase Reporter Assay

The activity of nuclear factor kappa-light-chain-enhancer of activated B cells (NF-*κ*B) was tested as previous description [24]. Briefly, the vector of pNF-*κ*B-Luc (Stratagene Inc., La Jolla, CA, USA) containing* Photinus pyralis* (firefly) luciferase reporter gene and the vector of phRL-TK vector (Promega, Madison, WI, USA) containing* Renilla* luciferase reporter gene were cotransfected into synovial fibroblasts using lipofectamine 2000 reagent. The transfected cells were simultaneously challenged with IL-1*β* and lunasin for 24 h. After treatment, the cell samples were collected and the activity of NF-*κ*B in cell samples was analyzed by TD-20/20 luminometer (Turner BioSystems, Sunnyvale, CA, USA) with a dual-luciferase reporter assay system (Promega, Madison, WI, USA).

### 2.10. Statistical Analysis

All experiments were repeated at least 3 times. Data were expressed as mean ± SD and analyzed by Student's *t*-test through GraphPad Prism Version 5.0 software (GraphPad Software Inc., San Diego, CA, USA). The statistically significant difference was considered at *P* < 0.05.

## 3. Results

### 3.1. Lunasin Inhibits the Proliferation of Synovial Fibroblasts

**Figure 1:**
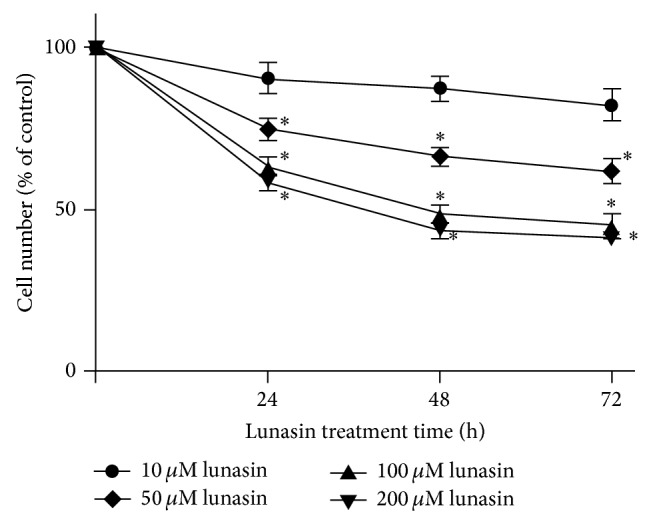
Lunasin inhibits the proliferation of RA synovial fibroblasts. Cells were synchronized and incubated with lunasin at various concentrations for 24, 48, and 72 h, respectively. Then, the cell samples were harvested and subjected to count the number by crystal violet staining. The IC_50_ was 153.3 ± 3.2 *μ*M after 48 h incubation. Data were expressed as the percentage relative to the untreated control samples, and each value was expressed as mean ± SD for three independent experiments by GraphPad Prism Software Version 5.0. ^*^ *P* < 0.05 was considered a significant difference when compared with the control.

The effect of lunasin on the proliferation of synovial fibroblasts was evaluated by crystal violet staining. As shown in Figure 1, lunasin significantly repressed the proliferation of synovial fibroblasts in a dose- and time-dependent manner. After 72 h incubation with lunasin at the concentrations of 100 *μ*M and 200 *μ*M, the cell number revealed a decrease by 44.7 ± 3.2% and 41.4 ± 2.0%, respectively, when compared with the control. In addition, lunasin treatment at a lower dose (50 *μ*M) for 24 h also could significantly inhibit the proliferation of synovial fibroblasts, whereas no obvious inhibition on cell proliferation was observed when synovial fibroblasts were treated with lunasin at the concentration of 10 *μ*M even for 3 days. The IC_50_ value of lunasin after 48 h treatment was 153.3 ± 3.2 *μ*M.

### 3.2. Lunasin Induces G0/G1 Phase Arrest of Synovial Fibroblasts

**Figure 2:**
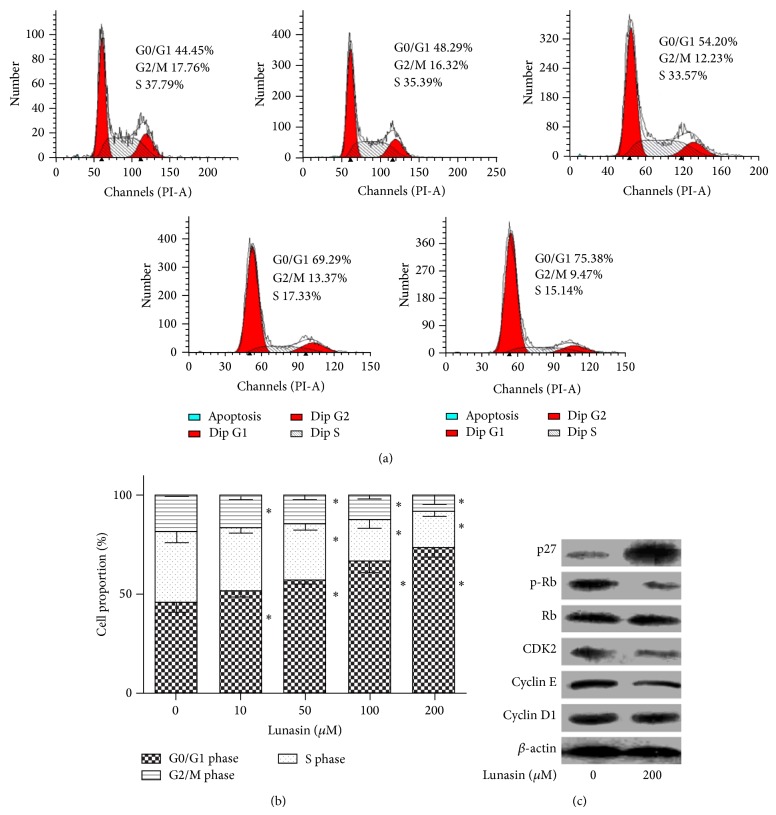
Effect of lunasin on the cell cycle of synovial fibroblasts and the expression of the proteins related to G0/G1 cell cycle arrest. (a) Cell cycle progression of synovial fibroblasts was examined by flow cytometry. Synovial fibroblasts were challenged with lunasin at concentrations of 0, 10, 50, 100, and 200 *μ*M for 48 h, respectively. After treatments, the cells were collected and subjected to the analysis of cell cycle distribution by flow cytometry. (b) Quantification of the cell cycle data. Data were expressed as mean ± SD for three independent experiments by GraphPad Prism Software Version 5.0. ^*^ *P* < 0.05 was considered a significant difference when compared with the control. (c) Synovial fibroblasts were treated with lunasin at the concentrations of 0 and 200 *μ*M for 48 h. The cell samples were harvested and the expression of cell cycle-relevant proteins was evaluated.

In order to explore whether the induction of cell cycle arrest contributes to the antiproliferative potency of lunasin in synovial fibroblasts, flow cytometric analysis was carried out to evaluate the cell cycle progression. As shown in Figures 2(a) and 2(b), the treatment of synovial fibroblasts with lunasin at gradually increasing concentrations (0, 10, 50, 100, and 200 *μ*M) for 48 h elevated the proportion of the cells in G0/G1 phase from 45.4 ± 2.5% up to 51.7 ± 2.0%, 59.2 ± 1.5%, 66.7 ± 3.2%, and 73.3 ± 2.4%, respectively, and on the contrary reduced the proportion of the cells in S phase from 35.8 ± 2.9% to 31.6 ± 1.6%, 29.1 ± 2.6%, 20.82 ± 2.2%, and 18.1 ± 1.5%, as well as decreasing the proportion of the cells in G2/M phase from 18.8 ± 0.6% to 16.7 ± 1.0%, 14.7 ± 1.3%, 12.6 ± 1.0%, and 8.9 ± 2.7%, respectively. These results indicated that lunasin arrested synovial fibroblasts in G0/G1 phase in a dose-dependent manner.

In order to further uncover the underlying mechanisms of lunasin on the inhibition of synovial fibroblast proliferation, we then examined the expressions of cell cycle-related proteins using Western blotting. Compared with the control, the treatment of synovial fibroblasts with 200 *μ*M lunasin for 48 h significantly decreased the expression of Cyclin-dependent kinase 2 (CDK2) and Cyclin E and conspicuously reduced the phosphorylation of Rb; in contrast, lunasin strongly elevated the level of p27 but failed to modify the production of Cyclin D1 (Figure 2(c)).

### 3.3. Lunasin Promotes the Apoptosis of Synovial Fibroblasts

**Figure 3:**
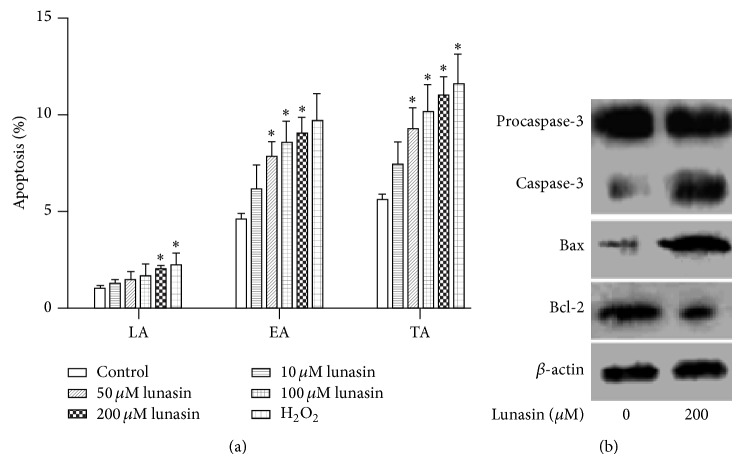
Effect of lunasin on the apoptosis of synovial fibroblasts and the expression of apoptosis-relevant proteins. (a) Synovial fibroblasts were incubated with lunasin at various concentrations (0, 10, 50, 100, and 200 *μ*M) for 48 h, and then the cells were harvested and the apoptosis proportion of the cells was examined by flow cytometry using V-FITC/PI double staining. The proportion of cells in early apoptosis (positive for FITC and negative for PI, FITC^+^/PI^−^) and late apoptosis (positive for both FITC and PI, FITC^+^/PI^+^) were analyzed by flow cytometry. Data were expressed as mean ± SD for three independent experiments. ^*^ *P* < 0.05 was considered a significant difference when compared with the control. (b) Synovial fibroblasts were treated with lunasin at the concentrations of 0 and 200 *μ*M for 48 h. The cell samples were then harvested and the expression of apoptosis-relevant proteins was evaluated. LA: late apoptosis; EA: early apoptosis; TA: total apoptosis.

In order to corroborate the possible suppression of cell proliferation by lunasin, synovial fibroblasts were double-stained with FITC and PI to examine the apoptosis by flow cytometry.  Figure 3(a) shows the quantitative data for the change in proportion of apoptotic cells. The cells exposed to lunasin revealed a clear apoptosis induction in a dose-dependent manner when compared with the control. After the cells incubated with lunasin at the concentrations of 0, 10, 50, 100, and 200 *μ*M for 48 h, the proportion of early apoptosis was enhanced from 4.6 ± 0.2% to 6.2 ± 0.7%, 7.8 ± 0.5%, 8.6 ± 0.6%, and 9.0 ± 0.5%, respectively, and the percentage of total apoptosis was elevated from 5.6 ± 0.2% to 7.4 ± 0.7%, 9.3 ± 0.6%, 10.17 ± 0.8%, and 11.0 ± 0.6%, respectively. These results suggest that lunasin is an effective inducer of apoptosis in synovial fibroblasts, especially for early apoptosis.

In order to elucidate the mechanisms of lunasin on the apoptosis of synovial fibroblasts, Western blotting was conducted to evaluate the change in the expression of apoptosis-relevant proteins. After synovial fibroblasts incubated with 200 *μ*M lunasin for 48 h, lunasin obviously induced the expression of activated caspase-3 and Bax; and suppressed the expression of antiapoptotic protein Bcl-2 (Figure 3(b)).

### 3.4. Lunasin Decreases the Production of IL-6, IL-8, and MMP-3 in RA Synovial Fibroblasts

**Figure 4:**
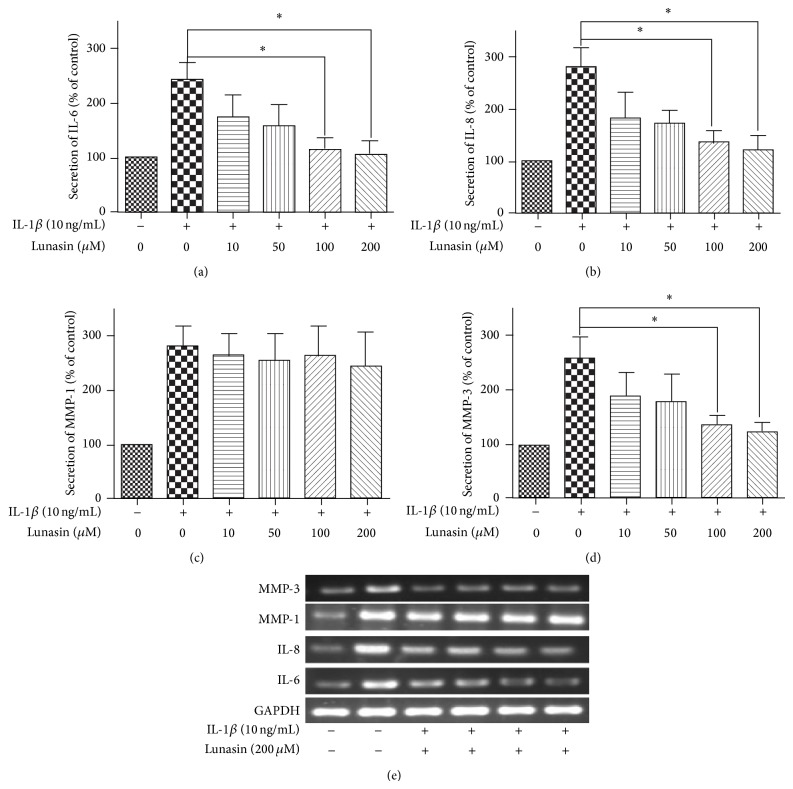
Effect of lunasin on the production of proinflammatory cytokines and MMPs. Synovial fibroblasts in the presence of IL-1*β* induction were treated with lunasin at various concentrations (0, 10, 50, 100, and 200 *μ*M) for 72 h in serum-free DMEM medium. After treatments, the cell-free medium and RA synovial fibroblasts were collected to evaluate the secretion of IL-6 (a), IL-8 (b), MMP-1 (c), and MMP-3 (d) by their corresponding ELISA kits, respectively. (e) The gene expression of IL-6, IL-8, MMP-1, and MMP-3 was examined by RT-PCR. Data were expressed as mean ± SD for three independent experiments by GraphPad Prism Software Version 5.0. ^*^ *P* < 0.05 was considered a significant difference when compared with the control.

The secretion of proinflammatory cytokines (IL-6, IL-8) and MMPs play an important role in RA progression. Therefore, we investigated the effect of lunasin on the secretion of IL-6, IL-8, MMP-1, and MMP-3. In the presence of IL-1*β* induction, the secretion of IL-6, IL-8, MMP-1, and MMP-3 revealed an obvious increase in the RA synovial fibroblasts. In contrast, the application of lunasin resulted in a dose-dependent reduction on the secretion of IL-6, IL-8, and MMP-3 in synovial fibroblasts induced by IL-1*β* (Figures 4(a)–4(c)); however, no obvious change in the secretion of MMP-1 was observed during the application of lunasin, even at the high dose (200 *μ*M) (Figure 4(d)).

To further confirm the effect of lunasin on the production of proinflammatory factors and MMPs in synovial fibroblasts, the gene expression of IL-6, IL-8, MMP-1, and MMP-3 was investigated by RT-PCR. In agreement with the observation of ELISA assay, IL-1*β* caused a markedly upregulated gene expression of IL-6, IL-8, MMP-1, and MMP-3; however, after the exposure to lunasin, synovial fibroblasts showed a significant decrease in the gene expression of IL-6, IL-8, and MMP-3 stimulated by IL-1*β*.

### 3.5. Lunasin Reverses IL-1*β*-Induced NF-*κ*B Activation in RA Synovial Fibroblasts

**Figure 5:**
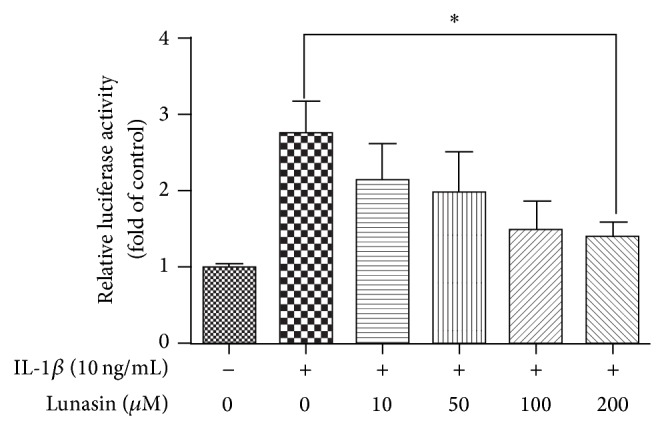
Lunasin inhibits the activation of NF-*κ*B in RA synovial fibroblasts in the presence of IL-1*β* induction. Transfected synovial fibroblasts were coincubated with IL-1*β* at the dose of 10 ng/mL and lunasin at the designed concentrations for 24 h. Then, the cell samples were collected and the activity of NF-*κ*B was analyzed. Data were mean ± SD for three independent experiments by GraphPad Prism Software Version 5.0. ^*^ *P* < 0.05 was considered a significant difference when compared with the control.

The activation of NF-*κ*B was observed in RA synovial fibroblasts and thought to be responsible for increasing the production of IL-6, IL-8, MMP-1, and MMP-3 [6–9]. Therefore, we investigated whether lunasin could affect the activation of NF-*κ*B. As shown in Figure 5, 10 ng/mL IL-1*β* caused a robust increase (2.73 fold) in NF-*κ*B activity, while lunasin revealed an inhibitory effect on the activation of NF-*κ*B in RA synovial fibroblasts in the presence of IL-1*β* induction.

## 4. Discussion

The hyperplasia of synovial tissue is ascribed to both an increased proliferation [25] and an impaired apoptosis of synovial fibroblasts [26]. The inhibition of synovial fibroblast proliferation is considered a potentially therapeutic approach for RA [27]. Traditional natural compounds or extracts from plants, for example, celastrol [10] or* Tripterygium wilfordii *Hook. F. extract [14], reveal their potential on the treatment of RA by inhibiting the proliferation RA synovial fibroblasts due to the induction of DNA damage, cell cycle arrest, and apoptosis; however, the high toxicity or unclear bioactive components in extracts largely limit their application. In the present study, we have found that lunasin from soybean without toxicity could significantly inhibit the proliferation of synovial fibroblasts through inducing G0/G1 phase arrests. We have also demonstrated that lunasin could promote the apoptosis of synovial fibroblasts in a dose-dependent manner. Lunasin contains a specific RGD sequence as a cell adhesion motif responsible for the attachment of lunasin to extracellular matrix, which has demonstrated the role of RGD peptide in inducing apoptosis in different cell lines via a caspase-dependent mechanism [28, 29]. Additionally, nine poly-D residues on the carboxyl termination of lunasin can specifically bind to histones, thereby affecting proper complex formation, leading to mitotic termination and eventually resulting in cell death [16], but these natural compounds including celastrol [10], sinomenine [11], and curcumin [12] extracted from plants or plant extracts containing similar small molecules such as* Tripterygium wilfordii *Hook. F. extract [14] can not accomplish the specific targeting function during the treatment process of RA. Previous investigations have reported that lunasin could induce the apoptosis of human colon cancer cells through the activation of mitochondrial pathway by upregulating Bax expression, decreasing Bcl-2 expression and promoting the activation of caspase-3 [30]. Since lunasin-induced apoptosis and G0/G1 cell cycle arrests in synovial fibroblasts are observed, we further investigated the effect of lunasin on the expression of proteins associated with G0/G1 cell cycle regulation and apoptosis modulation. In our study, Western blotting showed that lunasin significantly downregulated the expression of Cyclin E, CDK2 and suppressed the phosphorylation of Rb, as well as inhibited the expression of antiapoptotic protein Bcl-2 but elevated the production of p27, Bax, caspase-3, caspase-8, and caspase-9. Cyclin E is an important regulator of late G1 phase and its activity is positively correlated with its expression [31]. Rb becomes hyperphosphorylated after the activation of Cyclin E/CDK2; then, the phosphorylated Rb liberates E2F to generate the transcription of genes for the requirement of cell cycle progression and drives the cell into S phase [32]. P27 is a negative regulator of cell cycle and plays a crucial role in the inhibition of Cyclin/CDK complexes [33]. The data from Western blotting further confirm the inhibitory potency of lunasin on the proliferation of synovial fibroblasts.

Inflammatory changes of synovial fibroblasts also play a vital role in the progression of RA. It has been reported that IL-6 and IL-8 overexpressed in infected synovial fibroblasts. One data indicate that lunasin could inhibit the inflammation by inactivating NF-*κ*B pathway [34]. Moreover, the RGD peptide of lunasin has been considered to play an important role in suppressing inflammation [35]. In our study, we have also found that lunasin could significantly decrease the production of IL-6 and IL-8 in cultured RA synovial fibroblasts induced by IL-1*β* in a dose-dependent manner. IL-6 has been reported to inhibit the proliferation of synovial fibroblasts in the presence of soluble IL-6 receptor [36]. Contrarily, another observation reveals that IL-6 can induce the growth of synovial fibroblasts [37]. In our study, we have observed a conspicuous dose-dependent inhibition on the proliferation of synovial fibroblasts from RA patients after cells incubated with lunasin at gradually increasing concentrations. Moreover, we have observed that lunasin could decrease the secretion of IL-6 in RA synovial fibroblasts in the presence of IL-1*β* induction.

In addition, the cartilage destruction of RA is mostly caused by the activity of MMPs, and MMPs are pivotal in the recruitment of leukocytes and macrophages into joints [38]. Our results showed that lunasin suppressed the secretion of MMP-3 but did not affect the secretion of MMP-1. It is generally accepted that proinflammatory cytokines such as IL-1, TNF-*α*, and IL-6 are key mediators that greatly enhance the synthesis and secretion of MMPs [39]. In the present study, we have confirmed that lunasin can inhibit the secretion of IL-1*β*-induced IL-6 and IL-8 in RA synovial fibroblasts. Therefore, the reduced secretion of IL-6 and IL-8 induced by lunasin may be partly responsible for the decrease of MMP-3 secretion.

Mitogen-activated protein kinase (MAPK) and NF-*κ*B pathways are involved in regulating the expression of IL-6, IL-8, MMP-1, and MMP-3 in RA synovial fibroblasts [6, 8, 40]. According to one previous study, lunasin from soybean can inhibit the inflammation via the suppression of NF-*κ*B [34]. Consistent with other observations, our results clearly reveal that lunasin can suppress the activation of NF-*κ*B induced by IL-1*β* in RA synovial fibroblasts. However, there were no direct data to confirm the association of inhibiting NF-*κ*B activation and reducing proinflammatory cytokines, which needs to be further explored in the future.

RA is the one of the most autoimmune diseases worldwide and thus the development of effective strategies for the prevention and therapy of this disease is highly desired. Based on our comprehensive and systematic exploration in cultured RA synovial fibroblasts, lunasin may have promising potential for the development of a novel and effective nutritional supplement or drug candidate for RA through inhibiting the proliferation of RA synovial fibroblasts via apoptosis activation, suppressing the expression of proinflammatory cytokines, and reducing the production of MMPs in RA synovial fibroblasts. The further studies on lunasin treatment for RA in animal models and* in vivo* need to be conducted too in the future, which will provide the benefit for relieving the suffering of RA patients.

## Figures and Tables

**Table 1:** Primer sequences and reaction conditions of RT-PCR.

Genes	Primers	Sequences (5′-3′)	Annealing temperature (°C)	Cycle number
IL-6	Forward	GGCTGCTTCTGGTGATGG	55	30
	Reverse	AGAGATTTTGCCGAGGATGTA		

IL-8	Forward	GCCAAGGAGTGCTAAAGAACTTAGA	58	30
	Reverse	ATTTCTGTGTTGGCGCAGTGT		

MMP-1	Forward	AGGGTCAAGCAGACATCA	56	30
	Reverse	CAGAAGGGCAAGCATTAG		

MMP-3	Forward	CCTGCTTTGTCCTTTGATGC	55	30
	Reverse	TGAGTCAATCCCTGGAAAGTC		

GAPDH	Forward	CAAGGTCATCCATGACAACTTTG	56	25
	Reverse	GTCCACCACCCTG TTGCTGTAG		

## Abbreviations

RGD: Arginine-glycine-aspartic acid
CDK2: Cyclin-dependent kinase 2
MTT: 3-(4,5-Dimethylthiazol-2-yl)-2,5-diphenyltetrazolium bromide
DMEM: Dulbecco Modified Eagle Medium
ELISA: Enzyme linked immunosorbent assay
IL-6: Interleukin-6
IL-8: Interleukin-8
MMP: Matrix metalloproteinase
MAPK: Mitogen-activated protein kinase
NF-*κ*B: Nuclear factor kappa-light-chain-enhancer of activated B cells
PI: Propidium iodide
RA: Rheumatoid arthritis.
